# Evaluation of the Effects of Photodynamic Therapy Alone and Combined with Standard Antifungal Therapy on Planktonic Cells and Biofilms of *Fusarium* spp. and *Exophiala* spp.

**DOI:** 10.3389/fmicb.2016.00617

**Published:** 2016-04-27

**Authors:** Lujuan Gao, Shaojie Jiang, Yi Sun, Meiqi Deng, Qingzhi Wu, Ming Li, Tongxiang Zeng

**Affiliations:** ^1^Department of Dermatology, Zhongshan Hospital Fudan UniversityShanghai, China; ^2^Department of Gastroenterology, Jingzhou Central Hospital, The Second Clinical Medical College, Yangtze UniversityJingzhou, China; ^3^Department of Dermatology, Jingzhou Central Hospital, The Second Clinical Medical College, Yangtze UniversityJingzhou, China; ^4^The Second Clinical Medical College, Yangtze UniversityJingzhou, China

**Keywords:** photodynamic inactivation, *Fusarium*, *Exophiala*, planktonic, biofilm, antifungal susceptibility

## Abstract

Infections of *Fusarium* spp. *and Exophiala* spp. are often chronic, recalcitrant, resulting in significant morbidity, causing discomfort, disfigurement, social isolation. Systemic disseminations happen in compromised patients, which are often refractory to available antifungal therapies and thereby lead to death. The antimicrobial photodynamic therapy (aPDT) has been demonstrated to effectively inactivate multiple pathogenic fungi and is considered as a promising alternative treatment for mycoses. In the present study, we applied methylene blue (8, 16, and 32 μg/ml) as a photosensitizing agent and light emitting diode (635 ± 10 nm, 12 and 24 J/cm^2^), and evaluated the effects of photodynamic inactivation on five strains of *Fusarium* spp. and five strains of *Exophiala* spp., as well as photodynamic effects on *in vitro* susceptibility to itraconazole, voriconazole, posaconazole and amphotericin B, both planktonic and biofilm forms. Photodynamic therapy was efficient in reducing the growth of all strains tested, exhibiting colony forming unit-reductions of up to 6.4 log_10_ and 5.6 log_10_ against planktonic cultures and biofilms, respectively. However, biofilms were less sensitive since the irradiation time was twice longer than that of planktonic cultures. Notably, the photodynamic effects against *Fusarium* strains with high minimal inhibitory concentration (MIC) values of ≥16, 4-8, 4-8, and 2-4 μg/ml for itraconazole, voriconazole, posaconazole and amphotericin B, respectively, were comparable or even superior to *Exophiala* spp., despite *Exophiala* spp. showed relatively better antifungal susceptibility profile. MIC ranges against planktonic cells of both species were up to 64 times lower after aPDT treatment. Biofilms of both species showed high sessile MIC50 (SMIC50) and SMIC80 of ≥16 μg/ml for all azoles tested and variable susceptibilities to amphotericin B, with SMIC ranging between 1 and 16 μg/ml. Biofilms subjected to aPDT exhibited a distinct reduction in SMIC50 and SMIC80 compared to untreated groups for both species, except SMIC80 of itraconazole against *Fusarium* biofilms. In conclusion, *in vitro* photodynamic therapy was efficient in inactivation of *Fusarium* spp. and *Exophiala* spp., both planktonic cultures and biofilms. In addition, the combination of aPDT and antifungal drugs represents an attractive alternative to the current antifungal strategies. However, further investigations are warranted for the reliable and safe application in clinical practice.

## Introduction

Opportunistic fungi may causes cutaneous, subcutaneous and serious invasive infections, especially in immunocompromised and debilitated individuals. Invasive fungal infection represents a growing threat for human health due to difficulty in diagnosis and relatively few classes of available antifungal agents. *Fusarium* spp. and *Exophiala* spp. are both ubiquitous fungi commonly found in soil and on plants, and are increasingly recognized pathogen (Li et al., 2011; Guarro, 2013). *E. dermatitidis* is the leading cause of severe neurotropic phaeohyphomycosis (Li et al., 2011) and common cause of chromoblastomycosis; while fusariosis is, after aspergillosis, the second most common mold infection in humans, among which *F. solani* species complex and *F. oxysporum* species complex are responsible for approximately 60 and 20% of the cases, respectively (Guarro, 2013). Human infection usually occurs as a result of inoculation of the organism through the body surface causing local infection. Systemic dissemination, whose prevalence is effectively growing, occurs in subjects with underlying immunodeficiency (Li et al., 2011; Guarro, 2013), which is often refractory to available antifungal therapies and thereby leads to death (Filizzola et al., 2003; Guarro, 2013). Early management of local infection is crucial to prevent life-threatening disease. However, available antifungal drugs have shown poor *in vitro* activity against *Fusarium* spp. (Guarro, 2013). Fusariosis is mostly refractory to treatment, with a high mortality rate for systemic disseminations (Guarro, 2013). As for *Exophiala* spp. infection, success rate was only 40–70% although favorable *in vitro* activity of most antifungal drugs has been shown (Revankar and Sutton, 2010; Kondori et al., 2011; Patel et al., 2013). In addition to life-threatening infection, more frequently they result in significant morbidity, causing discomfort, disfigurement, social isolation, and they are usually recurrent and chronic.

Biofilm formation, which is a prerequisite event toward the development of invasive disease, has been reported to involve in about 80% of non-acute infections in human (Davies, 2003). Fungal biofilms, unlike planktonic forms, are relatively resistant to conventional antifungals, which may play an important role in the dissemination and therapeutic failure of *Fusaruim* and *Exophiala* infections (Desai et al., 2014). However, the eradication of biofilms is still a key challenge in the antifungal discovery agenda. Given that fungal infections always require lengthy antifungal therapy, alternative treatment methods are urgently needed.

In recent years, due to its efficiency and low invasive character, antimicrobial photodynamic therapy (aPDT) arises as a promising alternative approach to conventional antifungal medications, which has been demonstrated effective against multiple pathogenic fungi *in vitro* (Lyon et al., 2013; Pires et al., 2013; Arboleda et al., 2014; Morton et al., 2014; Nunes Mario et al., 2014; Baltazar et al., 2015) and applied successfully *in vivo* against cutaneous and subcutaneous mycoses (Gilaberte et al., 2011; Lyon et al., 2011b; Souza et al., 2014). In addition, aPDT could be a useful approach for the control of biofilms, and has been proposed for the growth control of oral candida (Pereira et al., 2011; Pereira Gonzales and Maisch, 2012). *In vitro* studies have shown that aPDT with methylene blue and light emitting diode (LED) was highly effective in killing *Foncecaea pedrosoi, Cladophialophora pedrosoi, Sporothrix schenckii* complex species et al. (Lyon et al., 2013; Nunes Mario et al., 2014). Lyon JP et al. employed methylene blue as photosensitizer and a LED device as light source, denoting the efficacy of aPDT *in vivo* against chromoblastomycosis (Lyon et al., 2011b). In this trial, an improvement of 90% of the clinical and histological aspect of the lesions was observed in all the 10 patients (Lyon et al., 2011b). Gilaberte et al. reported aPDT with 16% methylaminolevulinate cream and 635 nm LED successfully treated onychomycosis caused by the *F. oxysporum*, which was unresponsive to standard antifungals (Gilaberte et al., 2011). Despite these encouraging findings, aPDT revealed different effects against *Fusarium* spp. Rose bengal-mediated aPDT has been demonstrated to successfully inhibit the growth of *F. solani* (Arboleda et al., 2014). Pre-exposure to amphotericin B allowed riboflavin combined with long-wave ultraviolet effectiveness against *Fusarium* spp. (Sauer et al., 2010), while the combination of riboflavin and long-wave ultraviolet alone showed no antifungal effect on *F. solani* (Kashiwabuchi et al., 2013). In addition, little is known regarding the *in vitro* effects of aPDT on the growth and antifungal susceptibility of *Fusarium* spp. and the melanized pathogen *Exophiala* spp.

The aim of this study was to evaluate the effects of aPDT mediated by methylene blue with combination of LED on the viability of *in vitro* planktonic and biofilm forms of *Fusaruim* spp. and *Exophiala* spp., and to investigate the effects of aPDT on the antifungal susceptibilities.

## Materials and methods

### Fungal strains

Five strains of *E. dermatitidis* and 5 strains of *Fusarium* spp. (3 strains of *F. solani*, and 2 strains of *F. oxysporum*) were studied. All strains were clinical isolates and identified by molecular and morphologic methods. For the dermtermiantion of *in vitro* susceptibilities against antifungal agents, *Candida parapsilosis* ATCC 22019 was included to ensure quality control.

### Antifungal agents

All antifungal drugs including itraconazole (ITC; purity ≥ 99%), voriconazole (VRC; purity ≥ 99%), posaconazole (POS; purity ≥ 99%), and amphotericin (AMB; purity ≥ 80%) were purchased in powder form from Sigma Chemical Co., St. Louis, MO and prepared as outlined in the Clinical and Laboratory Standards Institute (CLSI) broth microdilution method M38-A2 (Institute, 2008). The working concentration ranges of tested drugs were all 0.06–16 μg/ml.

### Inoculum preparation

Conidia harvested from cultures grown for 7 days on Sabouraud dextrose agar (SDA) were suspended in sterile distilled water containing 0.03% Triton and diluted to a concentration of 1-5 × 10^6^ spores/ml. One milliliter of the suspension was added into 100 ml Sabouraud Dextrose broth. After incubation at 27°C in a shaker at 180 rpm for 48 h, conidia were collected and resuspended in saline solution to obtain a suspension of 1–5 × 10^6^ spores/ml.

### Biofilm preparation

Conidia were collected from SDA and resuspended in 20 ml Roswell Park Memorial Institute-1640 (RPMI-1640). RPMI-1640 without sodium bicarbonate supplemented with L-glutamine (Cellgro, cat. no. 50–020-PB) and buffered with 165 mM morpholinepropanesulfonic acid (Fisher, cat. no. BP308) to pH 7 is used for biofilm preparation according to the protocol (Pierce et al., 2008). The suspension was then adjusted to the final concentration of 1 × 10^7^ spores/ml. Subsequently, the suspension was added into the 96-well plate with 200 μl in each cell and incubated at 37°C for 48 and 72 h for *Fusarium* spp. and *Exophiala* spp., respectively. The media were then carefully extracted without disturbing the biofilm. The 96-well plate was washed with sterile PBS for three times to remove detached spores (Pierce et al., 2008).

### Photodynamic therapy of planktonic cultures

The photodynamic inactivation technique was described by Lyon et al. (2013), with modifications in the volume used, the incubation time, and the concentrations of methylene blue. The methylene blue was tested at concentrations of 8 μg/ml (T1), 16 μg/ml (T2), and 32 μg/ml (T3), with 100 μl of each concentration mixed with 100 μl of the standardized inocula prealiquoted into sterile 96-well microtiter plates. The suspensions were incubated for 2 h in the dark at 37°C. After this period, the inocula were irradiated using a LED with an irradiance of 100 mW/cm^2^ at a wavelength of 635 ± 10 nm and at a distance of 1 cm for 120 s (12 J/cm^2^). The following controls were included: fungal suspension in saline without irradiation (C1), fungal suspension with methylene blue (16 μg/ml) and without irradiation (C2), and fungal suspension in saline and irradiated (C3). After the irradiation period, 10 μl aliquots from each group were suspended with 90 μl saline, subsequently inoculated on SDA and incubated at 37°C for 48 and 120 h for *Fusarium* spp. and *Exophiala* spp., respectively. The viability of the conidia was then determined by counting colony-forming unit (CFU). All tests were performed in triplicate.

### Photodynamic therapy of biofilms

The methylene blue was also tested at concentrations as above, with 100 μl of each concentration added into 96-well plates containing biofilms. After incubation in dark for 2 h at 37°C, the biofilms were irradiated using a LED with an irradiance of 100 mW/cm^2^ at a wavelength of 635 ± 10 nm and at a distance of 1 cm for 240 s (24 J/cm^2^). The controls were included: biofilms in saline without irradiation (C1), biofilms with methylene blue (16 μg/ml) and without irradiation (C2), and biofilms in saline and irradiated (C3). After PDT treatment, 100 μl of sterile water was added into the well and washed vigorously in order to resuspend the biofilm cells thoroughly. The suspensions were then diluted 1000 times in sterile water and 100 μl aliquots were inoculated evenly on SDA. The number of CFU was determined after 24 and 48 h incubation at 37°C for *Fusarium* spp. and *Exophiala* spp., respectively. All tests were performed in triplicate.

### *In vitro* antifungal susceptibility of planktonic cultures

The individual minimal inhibitory concentrations (MICs) of ITC, VRC, POS, and AMB on photodynamic treated (T1) and untreated planktonic cells were determined according to M38-A2 method (Institute, 2008). The 96-well plate was inoculated with 100 μl of the inoculum suspension prepared and 100 μl of the serial diluent of tested drugs. Interpretation of results was performed after incubation at 35°C for 48 h for *Fusarium* spp. and 72 h for *Exophiala* spp., respectively. The MICs were determined as the lowest concentration resulting in complete inhibition of growth (Institute, 2008). All tests were performed in triplicate.

### *In vitro* antifungal susceptibility of biofilms

The 96-well plate was washed with sterile PBS for three times to remove detached spores. The individual sessile minimum inhibitory concentrations (SMICs) of ITC, VRC, POS and AMB on photodynamic treated (T1) and untreated *Exophiala* and *Fusarium* biofilms were assessed by the XTT {2,3-bis-(2-methoxy-4-nitro-5-sulfophenyl)-2H-tetrazolium-5-carboxanilide} based colorimetric assay (Ramage et al., 2001). After incubation at 37°C for 48 h, 100 μl XTT/menadione solution was added in each well and then incubated for another 4 h. Subsequently, 80 μl of the colored supernatant from each well was removed and transferred into a new plate, and read at 490 nm. The SMIC50 and SMIC80 was defined as the concentration at which a 50% or 80% decrease in optical density would be detected in comparison to the controls (Pierce et al., 2008). All tests were performed in triplicate.

### Analysis of results

For the purpose of analysis, CFU mL^−1^ values were transformed into logarithm (log_10_). The control group C1 was considered as 100% of growth for each set of tests. The photodynamic inactivation efficiency was evaluated by comparing the colony counts after treatments T1, T2, and T3 with those obtained with non-irradiated and methylene blue-free control colonies (C1).

The effect of photodynamic inactivation on the antifungal susceptibility was evaluated by comparing the MICs and SMICs of the photodynamic treated (T1) planktonic cultures and biofilms with those untreated, respectively.

## Results

### Photodynamic effects on the growth of planktonic cultures and biofilms

**Figure 1:**
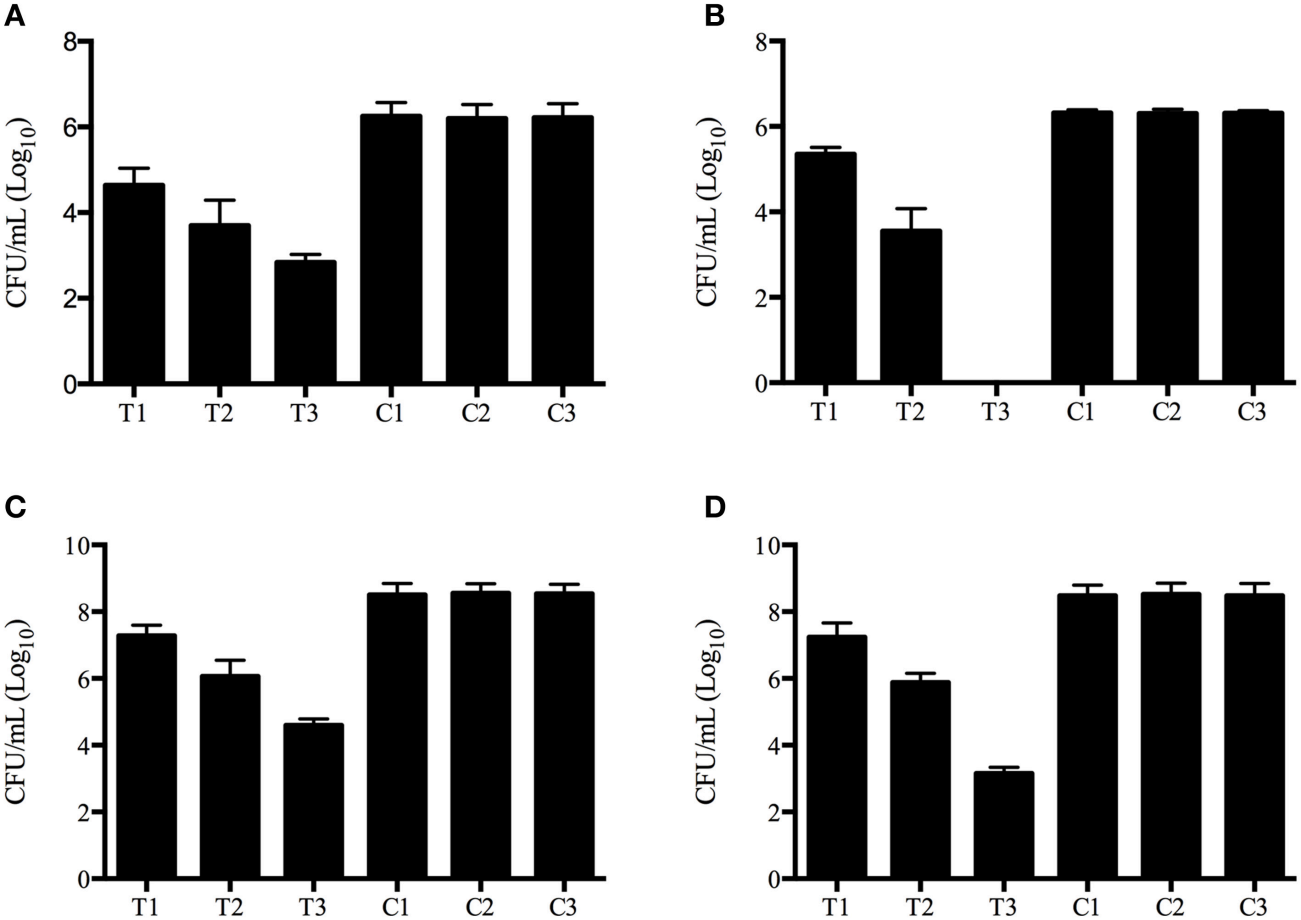
**Photodynamic inactivation effects on planktonic and biofilm forms of *Exophiala* spp. and *Exophiala* spp**. **(A)** CFU counting of Planktonic *Exophiala* spp. **(B)** CFU counting of Planktonic *Fusarium* spp. **(C)** CFU counting of *Exophiala* Biofilms. **(D)** CFU counting of *Fusarium* Biofilms. C1, growth in Sabouraud glucose agar without irradiation or methylene blue; C2, isolates exposed to 16 μg/ml of methylene blue without irradiation; C3 isolates exposed to irradiation without methylene blue; T1 photodynamic treatment with 8 μg/ml of methylene blue; T2 photodynamic treatment with 16 μg/ml of methylene blue; T3, photodynamic treatment with 32 μg/ml of methylene blue. Data are mean values and standard error from three replicate experiments.

The aPDT with methylene blue and LED exhibited CFU reductions of up to 3.8 log_10_ and 6.4 log_10_ against planktonic *Exophiala* spp. and *Fusarium* spp., respecitvely, and 4.2 log_10_ and 5.6 log_10_ against biofilms formed by *Exophiala* spp. and *Fusarium* spp., respecitvely, demonstrating efficacy in reducing the growth of both planktonic cultures and biofilms in all concentrations of methylene blue (Table 1 and Figures 1A–D).

**Table 1:** **Effect of photodynamic therapy on *Exophiala* spp. and *Fusarium* spp**.

**Strain**	**Planktonic**						**Biofilm**					
	**T1**	**T2**	**T3**	**C1**	**C2**	**C3**	**T1**	**T2**	**T3**	**C1**	**C2**	**C3**
*E. dermatitidis* (1)	2 × 10^5^	1.6 × 10^4^	1 × 10^3^	2 × 10^6^	1.8 × 10^6^	1.7 × 10^6^	1.5 × 10^7^	2 × 10^6^	4.2 × 10^4^	2.1 × 10^8^	2.4 × 10^8^	2.8 × 10^8^
*E. dermatitidis* (2)	5 × 10^4^	1 × 10^4^	1 × 10^3^	2.5 × 10^6^	2.2 × 10^6^	2.7 × 10^6^	2.5 × 10^7^	1 × 10^6^	6 × 10^4^	4.5 × 10^8^	3.8 × 10^8^	5.2 × 10^8^
*E. dermatitidis* (3)	3 × 10^4^	1.5 × 10^4^	8 × 10^2^	3 × 10^6^	2.8 × 10^6^	2.7 × 10^6^	3.2 × 10^7^	4.1 × 10^6^	5 × 10^4^	7.1 × 10^8^	7.9 × 10^8^	6 × 10^8^
*E. dermatitidis* (4)	2.5 × 10^4^	1 × 10^3^	4 × 10^2^	2.4 × 10^6^	2 × 10^6^	2.2 × 10^6^	5.8 × 10^6^	2 × 10^5^	1.9 × 10^4^	1 × 10^8^	1.5 × 10^8^	1.2 × 10^8^
*E. dermatitidis* (5)	2.1 × 10^4^	1.3 × 10^3^	5 × 10^2^	5 × 10^5^	4.3 × 10^5^	4.5 × 10^5^	3.4 × 10^7^	1.3 × 10^6^	4 × 10^4^	5.1 × 10^8^	5.5 × 10^8^	4.5 × 10^8^
*F. solani* (1)	3.2 × 10^5^	1.2 × 10^3^	0	2.7 × 10^6^	2.5 × 10^6^	2.5 × 10^6^	9 × 10^6^	3.5 × 10^5^	1.2 × 10^3^	1.1 × 10^8^	2.1 × 10^8^	1.2 × 10^8^
*F. solani* (2)	2.8 × 10^5^	2 × 10^4^	0	2.2 × 10^6^	2.4 × 10^6^	2 × 10^6^	3.9 × 10^7^	2 × 10^6^	2 × 10^3^	6.2 × 10^8^	6.6 × 10^8^	7.2 × 10^8^
*F. solani* (3)	2.7 × 10^5^	8 × 10^3^	0	1.8 × 10^6^	1.6 × 10^6^	2 × 10^6^	2.5 × 10^7^	7 × 10^5^	1 × 10^3^	4 × 10^8^	4.9 × 10^8^	4 × 10^8^
*F. oxysporum* (1)	1.6 × 10^5^	1.5 × 10^3^	0	2 × 10^6^	1.6 × 10^6^	2.1 × 10^6^	4 × 10^7^	6 × 10^5^	2.5 × 10^3^	5 × 10^8^	5.5 × 10^8^	5.8 × 10^8^
*F. oxysporum* (2)	1.4 × 10^5^	2 × 10^3^	0	1.8 × 10^6^	2.2 × 10^6^	1.7 × 10^6^	4.5 × 10^6^	8.2 × 10^5^	1 × 10^3^	1.9 × 10^8^	1.1 × 10^8^	1.2 × 10^8^

C1, growth in Sabouraud glucose agar without irradiation or methylene blue; C2, isolates exposed to 16 μg/ml of methylene blue without irradiation; C3 isolates exposed to irradiation without methylene blue; T1 photodynamic treatment with 8 μg/ml of methylene blue; T2 photodynamic treatment with 16 μg/ml of methylene blue; T3, photodynamic treatment with 32 μg/ml of methylene blue.

### Photodynamic effects on antifungal susceptibilities of planktonic cultures and biofilms

**Figure 2:**
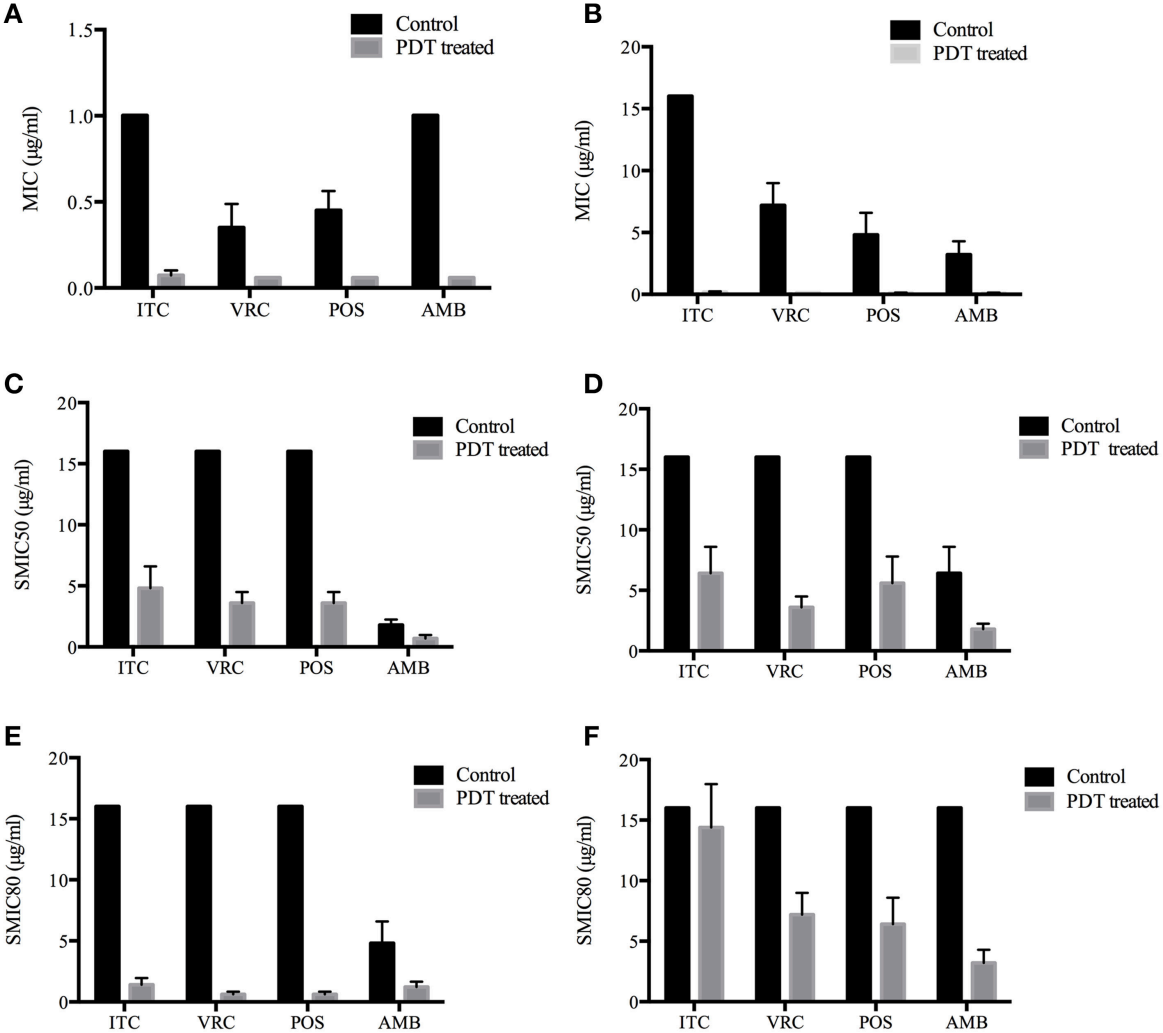
**Photodynamic effects on antifungal susceptibilities of planktonic and biofilm forms of *Exophiala* spp. and *Exophiala* spp. (A)** MICs of Planktonic *Exophiala* spp. **(B)** MICs of Planktonic *Fusarium* spp. **(C)** SMIC50 of *Exophiala* biofilm. **(D)** SMIC50 of *Fusarium* biofilm. **(E)** SMIC80 of *Exophiala* biofilm. **(F)** SMIC80 of *Fusarium* biofilm. ITC, itraconazole; VRC, voriconazole; POS, posaconazole; AMB, amphotericin B. Data are mean values and standard error from three replicate experiments.

MIC ranges of four drugs against planktonic cells with or without aPDT were summarized in Table 2. Planktonic *E. dermatitidis* without aPDT showed MIC values of 1 μg/ml for ITC and AMB, and 0.25–0.5 μg/ml for VRC and POS (Table 2). MICs of planktonic *E. dermatitidis* decreased to 0.125 μg/ml for ITC and 0.06 μg/ml for VRC, POS and AMB, respectively, after aPDT treatment, as shown in Figure 2A. Planktonic culture of *Fusarium* spp. without PDT showed high MIC values of ≥16, 4-8, 4-8, and 2-4 μg/ml for ITC, VRC, POS and AMB, respectively (Table 2). However, after aPDT treatment, the MIC ranges decreased to 0.125–0.25, 0.125, 0.06–0.125, and 0.06–0.125 μg/ml for ITC, VRC, POS, and AMB, respectively, as shown in Figure 2B.

**Table 2:** **Effect of photodynamic therapy on MICs of planktonic culture**.

**Strain**	**MICs (μg/ml)**							
	**Planktonic cultures**				**Planktonic cultures with T1 treatment**			
	**ITC**	**VRC**	**POS**	**AMB**	**ITC**	**VRC**	**POS**	**AMB**
*E. dermatitidis* (1)	1	0.5	0.5	1	0.06	0.06	0.06	0.06
*E. dermatitidis* (2)	1	0.25	0.5	1	0.06	0.06	0.06	0.06
*E. dermatitidis* (3)	1	0.25	0.5	1	0.125	0.06	0.06	0.06
*E. dermatitidis* (4)	1	0.25	0.5	1	0.06	0.06	0.06	0.06
*E. dermatitidis* (5)	1	0.5	0.25	1	0.06	0.06	0.06	0.06
*F. solani* (1)	≥16	8	4	4	0.125	0.125	0.125	0.06
*F. solani* (2)	≥16	4	4	2	0.25	0.125	0.125	0.125
*F. solani* (3)	≥16	8	4	4	0.125	0.125	0.125	0.125
*F. oxysporum* (1)	≥16	8	4	2	0.25	0.125	0.06	0.06
*F. oxysporum* (2)	≥16	8	8	4	0.125	0.125	0.125	0.125

SMIC ranges of four drugs against biofilms with or without PDT were summarized in Tables 3, 4. *Fusarium* and *Exophiala* biofilms showed high SMIC50 and SMIC80 of ≥16 μg/ml for all azoles tested. Both biofilms showed variable susceptibilities to AMB, with SMIC ranging between 1 and 16 μg/ml. Biofilms that were subjected to aPDT exhibited a distinct reduction in SMIC50 and SMIC80 compared to untreated groups for both species, except SMIC80 of ITC against *Fusarium* biofilms (Figures 2C–F).

**Table 3:** **Effect of photodynamic therapy on SMIC50 of biofilms**.

**Strain**	**SMIC50 (μg/ml)**							
	**Biofilm**				**Biofilm with T1 treatment**			
	**ITC**	**VRC**	**POS**	**AMB**	**ITC**	**VRC**	**POS**	**AMB**
*E. dermatitidis* (1)	≥16	≥16	≥16	2	4	4	4	0.5
*E. dermatitidis* (2)	≥16	≥16	≥16	1	4	4	2	0.5
*E. dermatitidis* (3)	≥16	≥16	≥16	2	8	4	4	0.5
*E. dermatitidis* (4)	≥16	≥16	≥16	2	4	2	4	1
*E. dermatitidis* (5)	≥16	≥16	≥16	2	4	4	4	1
*F. solani* (1)	≥16	≥16	≥16	4	4	4	8	2
*F. solani* (2)	≥16	≥16	≥16	8	8	4	4	2
*F. solani* (3)	≥16	≥16	≥16	4	8	4	4	1
*F. oxysporum* (1)	≥16	≥16	≥16	8	8	4	8	2
*F. oxysporum* (2)	≥16	≥16	≥16	8	4	2	4	2

**Table 4:** **Effect of photodynamic therapy on SMIC80 of biofilms**.

**Strain**	**SMIC80 (μg/ml)**							
	**Biofilm**				**Biofilm with T1 treatment**			
	**ITC**	**VRC**	**POS**	**AMB**	**ITC**	**VRC**	**POS**	**AMB**
*E. dermatitidis* (1)	≥16	≥16	≥16	8	2	0.5	0.5	2
*E. dermatitidis* (2)	≥16	≥16	≥16	4	1	0.5	0.5	1
*E. dermatitidis* (3)	≥16	≥16	≥16	4	2	1	1	1
*E. dermatitidis* (4)	≥16	≥16	≥16	4	1	0.5	0.5	1
*E. dermatitidis* (5)	≥16	≥16	≥16	4	1	0.5	0.5	1
*F. solani* (1)	≥16	≥16	≥16	≥16	16	8	8	4
*F. solani* (2)	≥16	≥16	≥16	≥16	8	4	4	2
*F. solani* (3)	≥16	≥16	≥16	≥16	16	8	4	4
*F. oxysporum* (1)	≥16	≥16	≥16	≥16	16	8	8	2
*F. oxysporum* (2)	≥16	≥16	≥16	≥16	16	8	8	4

## Discussion

Photodynamic inactivation combines the application of a pharmacologically inert chromophore, termed a photosensitizer (PS), and subsequent irradiation with visible light corresponding to the chromophore's specific absorption wavelength in the presence of molecular oxygen (Dai et al., 2012). After photon absorption the PS reaches an energized triplet state, which can undergo two mechanisms to regain its ground state. In type I mechanism, the PS directly transfers energy to a substrate or to molecular oxygen, producing reactive intermediates such as superoxide anion, hydrogen peroxide, hydroxyl radials, nitric oxide, and peroxide nitrite, while in type II mechanism, energy is transferred directly to molecular oxygen generating highly reactive singlet oxygen (Wainwright, 1998; Schweitzer and Schmidt, 2003; Hamblin and Hasan, 2004). The proportion of both mechanisms is unique for each PS with the singlet oxygen quantum yield Φ_Δ_ describing the proportion of type II mechanism (Maisch et al., 2007).

A variety of PSs have been used in antifungal photodynamic inactivation, including toluidine blue, methylene blue, Rose Bengal, porphyrins, phthalocyanines, 5-aminolevulinic acid, and curcumin (Calzavara-Pinton et al., 2012; Baltazar et al., 2015). A newly developed photosensitizer SAPYR, which exhibits a singlet oxygen quantum yield of 0.99 and absorption wavelength of 360–420 nm, has been demonstrated stronger effect against bacterial biofilms than methylene blue that exhibits a singlet oxygen quantum yield of 0.52 (Cieplik et al., 2015). Phthalocyanines are also characterized by high singlet oxygen quantum yields and high extinction coefficient in the far-red (680–720 nm) spectral region (Bertoloni et al., 1992; Calzavara-Pinton et al., 2012). A number of synthetic phthalocyanines, including chloro-aluminum phthalocyanine and silicon phthalocyanine 4, were demonstrated effective in *Candida albicans* and *Trichophyton rubrum in vitro* (Lam et al., 2011, 2014; Carmello et al., 2016).

However, when choosing a PS for antifungal photodynamic inactivation, the light penetration is an important concern. Given that fungal infections involve not only the superficial skin, but also the subcutaneous tissue, nails, hair, nasal cavity, oral cavity, esophagus or reproductive tract, some degree of light penetration is required (Donnelly et al., 2008). Moreover, dematiaceous fungi possess pigments (melanin), which could interfere with light absorption. Therefore, for melanized fungi such as *Exophiala* spp., the PS selected should absorb light in a different wavelength from that of the pigment present in the fungi. It is important to note that methylene blue have a absorption wavelength over 600 nm, which minimizes the competition with the melanin maximum absorption wavelength and allows maximal tissue transmission (Lyon et al., 2011a; Pires et al., 2014). Furthermore, the combination of methylene blue, which is already clinically approved for human use, and LED is a very inexpensive and convenient system, and is increasingly being used in experimental and clinical applications of aPDT(Calzavara-Pinton et al., 2012; Dai et al., 2012). We therefore chose to apply aPDT using methylene blue and LED irradiation for studying the aPDT effects against planktonic and biofilm forms of *Fusaruim* spp. and *Exophiala* spp. in an *in vitro* assay.

The results in the present study revealed that all isolates tested were sensitive to photodynamic inactivation, both planktonic cells and biofilms. Planktonic and biofilm form of *Exophiala* spp. exhibited CFU reductions of up to 3.8 log_10_ and 4.2 log_10_, respectively, which is declared as biologically relevant antimicrobial activity (Boyce et al., 2002). Planktonic and biofilm form of *Fusarium* spp. exhibited CFU reduction of up to 6.4 log_10_ and 5.6 log_10_, respectively, which is defined as disinfect effect (Boyce et al., 2002) and has not been reported in the literature thus far. Previous study has shown in *C. albicans* that ATP-binding cassette, a multidrug efflux system was directly implicated in methylene blue efflux from the cell cytoplasm, which might impact the antimicrobial photodynamic inactivation efficacy (Prates et al., 2011). However, in this study, *Fusarium* strains with high MIC values to ITC, VRC and POS were also sensitive to aPDT mediated by methylene blue. Despite *Exophiala* spp. showed relatively better antifungal susceptibility profile, the inactivation rates of planktonic and biofilm form of *Fusaruim* spp. in all treatment groups were comparable or even superior to *Exophiala* spp., suggesting that aPDT is active regardless of antifungal resistance, as described previously (Mima et al., 2010). This might relate to the principles of the action of nonspecific oxidizing agents of PDT, which oxidize biological molecules of the fungi cells in multiple targets (Lyon et al., 2011a).

Compared to planktonic cells, biofilms were less sensitive to aPDT since the irradiation time of biofilms was twice longer than that of planktonic cultures. Previously, biofilms have been found to be more resistant to aPDT than planktonic cells in several studies (Donnelly et al., 2007; Chabrier-Roselló et al., 2008; Dovigo et al., 2011; Costa et al., 2012). Therefore, longer preirradiation time or higher PS concentrations are needed to obtain better aPDT response (Donnelly et al., 2007; Chabrier-Roselló et al., 2008; Dovigo et al., 2011; Costa et al., 2012). In this study, we were able to achieve satisfactory photodynamic effects by double the irradiation time of the biofilms. This difference probably occurred due to the structural characteristics of biofilms, including restriction of penetration by the extracellular matrix, the decreased growth rate and heterogeneity of the biofilm cells, and distinct gene expression levels (Costa et al., 2012).

We also tested the change of antifungal susceptibilities of tested strains before and after aPDT treatment, in both planktonic and biofilm forms. Pre-treatment with aPDT followed by standard antifungal treatments resulted in dramatic reduction of MICs and SMICs for both species. Both planktonic suspensions and biofilms were much more susceptible to antifungal drug treatments after aPDT, which may due to the increased membrane permeability caused by photodynamic inactivation, as demonstrated in previous study (Giroldo et al., 2009). The results suggest aPDT combined with standard antifungal treatment may help to enhance the antifungal susceptibility to overcome problems with drug resistance issues, and has the potential to reduce drug dosages and drug toxicity.

In general, our results expand the knowledge regarding the photodynamic inactivation of pathogenic fungi. The *in vitro* photodynamic therapy with methylene blue and LED was efficient in inhibiting the growth of *Fusaruim* spp. and *Exophiala* spp., both planktonic cultures and biofilms. In addition, the combination of aPDT and antifungal drugs represents an attractive alternative to the current antifungal strategies for infections of *Fusaruim* spp. and *Exophiala* spp., which has the potential to reduce treatment times, drug dosages, drug toxicity and improve patient compliance. However, despite the encouraging results, further investigations including *in vivo* experimental and clinical studies are warranted to determine clear protocols for the reliable and safe application in clinical practice.

## Author contributions

LG, SJ, and YS conceived and designed the study. SJ, MD, and QW performed all the experiments. LG and YS analyzed the data and wrote the manuscript. ML and TZ provided general guidance and revised the manuscript.

## Funding

This work was supported by grants 31400131 (LG) and 81401677 (YS) from National Natural Science Foundation of China, grant 2015ZSYXQN21 from Outstanding Youth Project of Zhongshan Hospital Fudan University (LG) and grant WJ2015MB281 from Hubei Province Health and Family Planning Scientific Research Project (YS).

### Conflict of interest statement

The authors declare that the research was conducted in the absence of any commercial or financial relationships that could be construed as a potential conflict of interest.
